# Genomics of Treatable Traits in Asthma

**DOI:** 10.3390/genes14091824

**Published:** 2023-09-20

**Authors:** Antonio Espuela-Ortiz, Elena Martin-Gonzalez, Paloma Poza-Guedes, Ruperto González-Pérez, Esther Herrera-Luis

**Affiliations:** 1 Genomics and Health Group, Department of Biochemistry, Microbiology, Cell Biology and Genetics, Universidad de La Laguna (ULL), 38200 San Cristóbal de La Laguna, Tenerife, Spain; aespuela@ull.edu.es (A.E.-O.); emarting@ull.edu.es (E.M.-G.); 2 Allergy Department, Hospital Universitario de Canarias, 38320 Santa Cruz de Tenerife, Tenerife, Spain; pozagdes@hotmail.com (P.P.-G.); glezruperto@gmail.com (R.G.-P.); 3 Severe Asthma Unit, Hospital Universitario de Canarias, 38320 San Cristóbal de La Laguna, Tenerife, Spain; 4 Department of Epidemiology, Bloomberg School of Public Health, Johns Hopkins University, Baltimore, MD 21205, USA

**Keywords:** asthma, GWAS, genomics, precision medicine, asthma phenotype, endotype

## Abstract

The astounding number of genetic variants revealed in the 15 years of genome-wide association studies of asthma has not kept pace with the goals of translational genomics. Moving asthma diagnosis from a nonspecific umbrella term to specific phenotypes/endotypes and related traits may provide insights into features that may be prevented or alleviated by therapeutical intervention. This review provides an overview of the different asthma endotypes and phenotypes and the genomic findings from asthma studies using patient stratification strategies and asthma-related traits. Asthma genomic research for treatable traits has uncovered novel and previously reported asthma loci, primarily through studies in Europeans. Novel genomic findings for asthma phenotypes and related traits may arise from multi-trait and specific phenotyping strategies in diverse populations.

## 1. Introduction

Asthma is a major noncommunicable, respiratory disease that affects an estimated 350 million people worldwide and is the most prevalent chronic disease in children globally [1]. It is a heterogeneous and complex disease, characterized by chronic airway inflammation and a history of respiratory symptoms such as wheeze, shortness of breath, chest tightness, and cough that vary over time and in intensity, together with variable expiratory airflow limitation [2]. Different environmental, genetic, and behavioral factors interact to modify asthma’s susceptibility and course, which contributes to the disease complexity [2]. Regrettably, despite asthma mortality having decreased in the last decades, still a substantial proportion of asthmatics remain difficult to treat, leading to significant economic consequences, including productivity losses and increased cost of public health expenditure [3,4,5].

Early genetic studies of asthma had limited success in associating genetic variation with asthma susceptibility using linkage analyses in large families with more than one person with asthma, as well as using candidate gene association analyses. Novel genetic signals arose with the advent of genome-wide association studies (GWAS), which are hypothesis-free scans that interrogate genetic variation across the genome for association with a phenotype of interest. Despite GWAS having revealed a large catalog of genetic loci for asthma, the genetic variation uncovered only accounts for a small fraction of asthma heritability, with higher contribution to childhood-onset asthma (33%) than to adult-onset asthma (9.8%), as found in British individuals [6]. Genomic research has investigated several asthma phenotypes or asthma-related traits in an attempt to unravel the complicated etiologic pathways of asthma and features that could be prevented or alleviated by therapeutic interventions such as pulmonary rehabilitation or pharmacological treatment. Here, we provide an overview of the different asthma endotypes and phenotypes along with their clinical characteristics, clinically relevant markers, and molecular mechanisms. We also provide an update on GWAS findings across asthma phenotypes and related traits to identify strategic research opportunities for treatable traits moving forward asthma precision medicine.

## 2. Asthma Endotypes and Phenotypes

The asthma definition has largely evolved from the early clinical descriptions by Dr. Henry Hyde Salter in the 19th Century [7] to the current understanding of this heterogeneous disease as an umbrella term comprising numerous and different asthma subtypes [8]. A prevailing approach to categorize asthma has been to group patients on observable attributes arising from a complex interplay between hereditary, environmental, and behavioral influences. In fact, the first approach to asthma phenotyping was documented in the late 1940s when Rackemann distinguished between extrinsic—atopic—and intrinsic—unrelated to atopy—asthma [9], and skin tests were often helpful in confirming diagnosis and determining a specific treatment [10]. Since 1999, the clinical and physio-pathological characterization of severe asthmatics—according to the number of eosinophils in the airway—has subsequently inspired a myriad of studies aiming to discriminate between eosinophilic (EA) and non-eosinophilic asthma (NEA). In 2006, Hinks and colleagues assessed the proportion of eosinophils and neutrophils in induced sputum, depicting four asthma phenotypes—EA, NEA, paucigranulocytic asthma (PGA), and mixed-granulocytic asthma (MGA) [11]. Furthermore, gene expression analysis confirmed in 2009, two distinct asthma subgroups—Th2-high and Th2-low—defined by the degree of underlying Th2 inflammation and regardless of patients’ demographic characteristics, lung function, or bronchodilator response [12]. Thus, the definition of the Th2-high asthma phenotype was initially based on atopic predisposition in combination with any of the following surrogate biomarkers for Th2 immune activation: serum immunoglobulin E (IgE) ≥ 100 IU/mL, blood eosinophil count ≥ 300/μL, and exhaled nitric oxide fraction (FENO) ≥ 30 ppb [13]. However, since the production of Th2-related cytokines such as interleukins 4, 5, and 13 (IL-4, IL-5, and IL-13) has been confirmed in further cell populations as type 2 innate lymphoid cells (ILC-2s), mast cells, basophils, and/or eosinophils, the term Th2 has been currently updated to the T2 immune phenotype in asthma [14]. Notably, some of these cytokines may also affect cell counts in asthmatics (i.e., IL5-promoted eosinophilia) [14]. Conventional asthma phenotyping classifies patients according to observable clinical features, including exacerbating factors, age of onset, concomitant comorbidities, and/or response to therapy [15]. As these clinical categories could not discriminate among groups or elucidate the underlying pathobiology, multivariate statistical cluster analysis performed on large asthma cohorts such as SARP [16], U-BIOPRED [17], or ADEPT [18] have greatly contributed to the unbiased description of specific asthma phenotypes [19]. Despite differences in clusters being found, two major groups, namely type 2 (T2)-high and non-T2-high, have been currently defined [20]. These evolving endotypes—associating plausible molecular and cellular mechanisms or therapeutic response to phenotypes—have, nowadays, pioneered asthma into the age of precision medicine [21,22].

### 2.1. T2-High Asthma

In T2-high asthma, the interaction of the airway epithelium with the external exposome activates the release of specific mediators—epithelial-derived alarmins—as thymic stromal lymphopoietin (TSLP), IL-25, and IL-33, leading to the production of IL-4, IL-5, and IL-13 [23]. Subsequent tT2 immuno-responses include IgE-mediated hypersensitivity to aeroallergens, chemoattraction of mast cells, eosinophils, and basophils, and remodeling of the airway epithelium [14]. T2-high asthma has been clinically classified into three phenotypes, including early-onset allergic asthma, late-onset eosinophilic asthma, and nonsteroidal anti-inflammatory drugs (NSAIDs)-exacerbated respiratory disease (NERD) [20,24].

#### 2.1.1. Early-Onset Atopic Asthma

The early-onset atopic asthma phenotype—most frequently identified in former hierarchical clustering analysis—is predominant in children, responsive to inhaled steroids, and commonly associated with increased T2 cytokines, serum-specific IgE to inhalants, and allergic comorbidities, i.e., allergic rhinitis, atopic dermatitis, and/or food allergy, with a relevant participation in the “atopic march” [8,25]. Multiple environmental factors, including allergens, viral infections, pollutants, and/or cigarette smoke, have been described as potential triggers to activate inflammatory responses, leading to clinical symptoms concerning this asthma phenotype [26,27,28]. Despite asthma symptoms—with variations in severity—that are elicited during childhood and may be resolved in adolescence, this phenotype can persist through life [19,29].

#### 2.1.2. Late-Onset Eosinophilic Asthma

Late-onset eosinophilic asthma phenotype usually starts in adulthood, and its underlying pathobiology is also driven by a preponderant T2 inflammation response with apparently no evidence of atopy but the leading role of ILC-2 in the production of IL-5 and IL-13 [30]. Although this phenotype may show different clinical presentations, including comorbid chronic rhinosinusitis with and without nasal polyps, a significant proportion of patients are older and have a more severe disease, lower pulmonary function, increased blood and sputum eosinophils, and are partially responders to inhaled or systemic steroids [31,32].

#### 2.1.3. Nonsteroidal Anti-Inflammatory Drugs-Exacerbated Respiratory Disease (NERD)

NERD is considered as a subset of the late-onset eosinophilic asthma phenotype—frequently associated with chronic rhinosinusitis with nasal polyps (CRSwNP)—presenting with rapid respiratory exacerbations immediately triggered after the intake of aspirin or other NSAID drugs that inhibit the cyclooxygenase-1 isoenzyme (COX-1). Despite the complete underlying pathogenic mechanism remaining unclear, NERD is characterized by a dysregulation in the arachidonic acid metabolism and a marked overproduction of cysteinyl leukotrienes (cysLTs), a potent lipid inflammatory mediator derived from arachidonic acid [33,34]. Mast cells, eosinophils and platelet-adherent leukocytes, which are present in the respiratory tissue of subjects with NERD have functional 5-lipoxygenase (5-LOX) and leukotriene (LT) C4 synthase enzymes [35]. Arachidonic acid is oxidized by 5-LOX to form short-lived LT mediators, such as LTC4, LTD4, and the stable metabolite LTE4 that has been formerly described as a biomarker in patients with NERD [36,37,38]. Interestingly, innate type 2 mediators from epithelial cells can be also activated after stimulation with cysLTs and further amplified by mast-cell-derived prostaglandin D2 gene (*PGD2*), leading to the persistent eosinophilic airway inflammation, bronchoconstriction, and mucus secretion related to refractory nasal polyposis and asthma [39,40].

### 2.2. T2-Low Asthma

Clinically, T2-low asthma—accounting for 33 to 50% of the asthmatics—has been grouped according to obesity, smoking exposure, and age. T2-low asthma is characterized by the activation of non-T2 inflammatory pathways, including helper T-lymphocytes type 1 (Th1) and/or Th17 cells, IL-6, IL-8, IL-17, and IL-22, and epithelial-derived cytokines [41,42]. Despite no validated biomarkers having been confirmed yet, sputum cytology has defined different subsets for T2-low asthma: neutrophilic (sputum neutrophils > 40–60%) and paucigranulocytic (normal sputum levels of neutrophils and eosinophils) asthma [43]. Patients with T2-low asthma usually develop symptoms at adulthood, and they are frequently associated with obesity, cigarette smoke exposure, lower bronchodilator reversibility, chronic infection with atypical bacteria, and a limited response to inhaled and systemic steroids in combination with a metabolic dysfunction [44,45,46]. Comorbidities such as hypertension and diabetes are frequent in this subset of patients with lower lung function and increased blood IL-6 levels, which has been considered a putative biomarker for metabolic dysfunction [22,47].

PGA has been identified as a milder respiratory phenotype in terms of severity, number of clinically relevant exacerbations, and improved lung function compared to EA and neutrophilic asthma (NA) [48]. Patients with PGA show lower levels of biomarkers of both eosinophilic—blood and sputum eosinophils, serum periostin, eosinophilic cationic protein (ECP), and FENO—and neutrophilic inflammation—serum matrix metalloproteinase-9 (MM-9), and IL-8 [48,49]. Despite the immunopathological underlying mechanisms not having been elucidated yet, PGA is characterized by increased airway smooth muscle dysfunction—hyperplasia and hypertrophy—leading to chronic airflow obstruction and release of inflammatory mediators due to specific neurogenic pathways [50,51]. As no biological treatment is available for T2-low asthma, alternative therapy targeting airway smooth muscle dysfunction including mitogen-activated protein kinase inhibitors, tyrosine-kinase inhibitors, phosphatidylinositol 3 kinase inhibitors, or phosphodiesterase inhibitors is currently under investigation [52,53,54].

#### 2.2.1. Obesity-Associated Asthma

Obesity-associated asthma is a complex asthma phenotype more frequently described in nonatopic middle-aged females, presenting with severe respiratory symptoms and a relatively preserved pulmonary function [55,56]. Interestingly, the inflammatory response in obesity is associated with a switch from Th2 cells to Th1, Th17, and cytotoxic T lymphocytes [57]. In addition, the levels of specific cytokines have been positively related to body-mass index (BMI) [58]. Further innate inflammatory pathways involving ILC-3s expressing IL-17 and IL-22 have been also described in obesity-associated asthma [59]. The proinflammatory cytokine IL-6, produced in adipocytes and adipose tissue macrophages, has been associated with obese T2-low asthma but not with obese atopic asthma [59,60]. Moreover, a reduction in arginine and nitric oxide (NO) bioavailability has been related to the increased oxidative stress occurring both in obesity and obese adults with the late-onset asthma phenotype [61].

#### 2.2.2. Smoking-Associated Asthma Phenotype

The estimated prevalence of smokers within asthmatics—about 20%—is similar to that found in the general population [62,63]. Cigarette smoking in asthmatics has been previously related to poor control of symptoms, increased mortality, declined pulmonary function, lower response to steroids, and increased healthcare costs [64,65,66]. The recognition of a smoking-associated asthma phenotype has relevant implications to an improved management of patients afflicted with this specific asthma subtype. In this regard, smoking-associated asthma is considered a T2-low neutrophilic phenotype speculating that persistent exposure to cigarette smoke may induce a predominance of activated macrophages producing proinflammatory molecules, reactive oxygen species, matrix metalloproteinases, and specific chemokines such as IL-8, contributing to the prolonged survival of neutrophils in the lung tissue [67]. In addition, cigarette smoke increases total IgE levels and the risk of sensitization to aeroallergens, thus enhancing a combined Th1/Th2 inflammatory response developing a more severe asthma phenotype and a putative link between asthma and chronic obstructive pulmonary disease (COPD) in subjects with a relevant smoking history, airflow obstruction, and overlapping features of asthma, termed asthma–COPD overlap syndrome (ACO) [53,68,69].

#### 2.2.3. Elderly-Associated Asthma Phenotype

The age cutoff value in this underdiagnosed and sub-optimally treated very-late-onset asthma phenotype is >65 years [70]. Age-related changes in the lung structure such as airway narrowing, reduced elastic recoil, or alveolar dilation may lead to an overall decreased pulmonary function [71,72]. Although the pathobiology of this phenotype has not been totally elucidated the preponderant airway neutrophilic inflammation has been related to both Th1 and Th17 responses [73,74].

### 2.3. Overlapping in Asthma Phenotypes

An elevated rate of overlapping—above 70%—has been described in mild-to-severe asthmatics, including combinations among different inflammatory asthma phenotypes, such as T2-high, T2-low, and mixed T2/non-T2 [75] (Table 1). In this regard, occupational asthma (OA), a subtype of work-related asthma, currently shows as a challenging respiratory model to clinical phenotyping. As both high-molecular-weight (HMW) proteins and low-molecular-weight (LMW) chemicals can elicit OA, different clinical, physiological, and inflammatory profiles have been described, with HMW agents showing a higher baseline blood eosinophilia and a greater post-challenge elevation in associated FENO levels [76,77]. In fact, asthma has been proposed as a nonlinear complex dynamic system with both clinical and therapeutic implications, suggesting an evolution in the underlying inflammatory status from an initial T2-high profile moving towards an alternative T2-low or mixed T2/non-T2 asthma phenotype [78,79,80].

## 3. Genomic Studies

**Figure 1:**
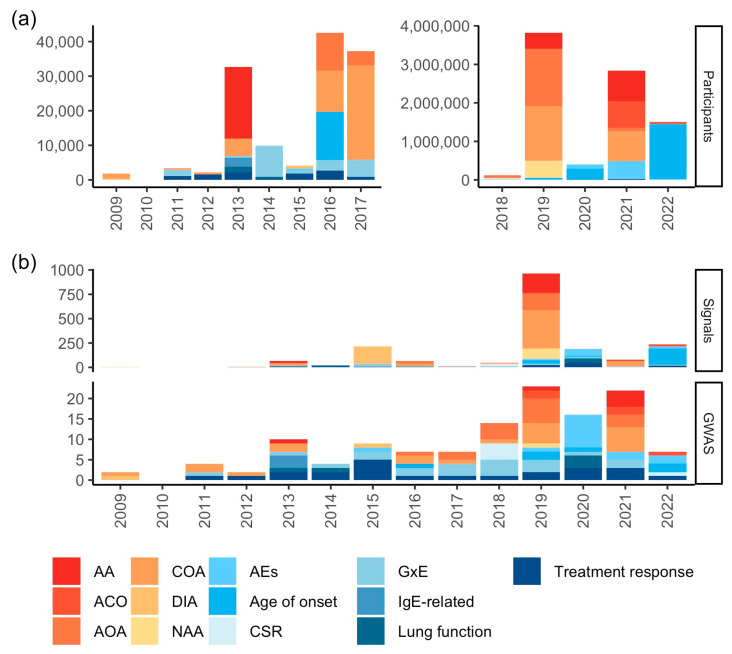
Characteristics and findings from GWAS of asthma phenotypes and related traits over time, 2009–2022, as curated by the NHGRI-EBI GWAS Catalog. (**a**) Maximum number of participants in the discovery GWAS stage; (**b**) number of associations and GWAS. Abbreviations: AA: allergic asthma; ACO: asthma–chronic obstructive pulmonary disease overlap syndrome; AEs: asthma exacerbations; AOA: adult-onset asthma; COA: childhood-onset asthma; CSR: asthma control, severity, or remission; DIA: diisocyanate-induced asthma; GxE: gene–environment interaction; IgE: immunoglobulin E; NAA: nonatopic asthma.

Among genomic studies, GWAS have uncovered a plethora of associations for several diseases and complex human traits and diseases, mainly comprising common genetic variants (usually with a minor allele frequency (MAF) ≥ 1%) of low-to-moderate effect sizes (~0.8 < odds ratio (OR) < ~1.3 for most asthma-related variants) [6,81]. GWAS findings regarding asthma phenotypes and related traits were characterized by querying the latest version of the NHGRI-EBI GWAS Catalog [82] as detailed in Appendix A. As of 7 April 2023, the NHGRI-EBI GWAS Catalog encompasses 31 publications related to asthma phenotypes spanning 52 unique study accession numbers—trait-specific analyses conducted within each publication—across 24 unique outcomes and 973 unique associations. In addition, a total of 51 publications of asthma-related traits comprised 76 unique study accession numbers, 61 unique outcomes, and 464 unique associations. Overall, the outburst of GWAS with a growing number of participants has risen the number of identified genetic signals, except in 2021, when the analysis of more than 2.8 million individuals revealed less than 80 associations across outcomes (Figure 1a,b). The maximum number of GWAS participants included in the discovery stage for an asthma phenotype was 601,193 (for childhood-onset asthma (COA) in Europeans and Japanese [83]), while it amounts up to 730,758 for asthma-related trait findings (specifically, for age of onset in ethnically diverse individuals [84]).

**Figure 2:**
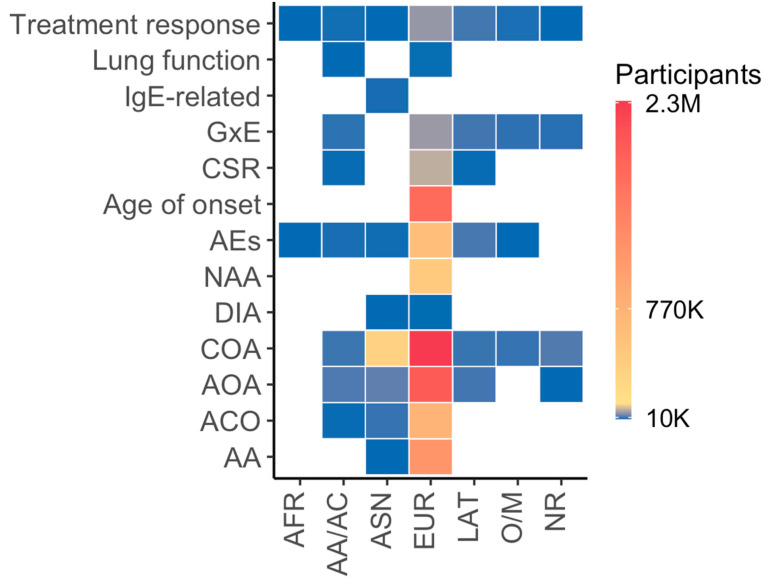
Outcome distribution per “broad” ancestry category and number of participants in GWAS of asthma phenotypes and related traits over time, 2009–2022, as curated by the NHGRI-EBI GWAS Catalog. Abbreviations: AA: allergic asthma; ACO: asthma–chronic obstructive pulmonary disease overlap syndrome; AA/AC: African American or Afro-Caribbean; AEs: asthma exacerbations; AFR: African; AOA: adult-onset asthma; ASN: Asian; COA: childhood-onset asthma; CSR: asthma control, severity, or remission; DIA: diisocyanate-induced asthma; EUR: European; GxE: gene–environment interaction; IgE: immunoglobulin E; LAT: Hispanic/Latin American; NAA: nonatopic asthma; NR: not reported; O/M: Other/Mixed.

Asthma phenotypes have been widely investigated in Europeans compared with other ancestry groups and asthma-related traits (Figure 2), largely due to the contribution of the population-based United Kingdom Biobank (UKB). Furthermore, asthma-related traits remain widely unexplored across diverse populations, partially because these data might have not been extensively collected in population-based studies. Notably, African populations are poorly represented in both GWAS of asthma phenotypes and related traits. Nevertheless, African-admixed populations, mainly African Americans, have been included in genetic studies of asthma phenotypes and related traits for which African Americans exhibit differential profiles in comparison with other ancestry groups (e.g., asthma exacerbations, treatment response, or lung function). Across all outcomes, lung function, IgE-related phenotypes, asthma control, severity or remission, age of asthma onset, and nonatopic asthma are the least investigated across ancestry groups. Conversely, asthma exacerbations and treatment response have been investigated across most of the ancestry groups, despite the modest sample sizes compared to GWAS of asthma phenotypes in Europeans. Similarly, gene–environment interactions still lag behind compared to GWAS of asthma phenotypes, possibly due to the even large sample size required to detect interaction signals after multiple comparison testing (Figure 2).

### 3.1. Genomic Studies of Asthma Phenotypes

**Figure 3:**
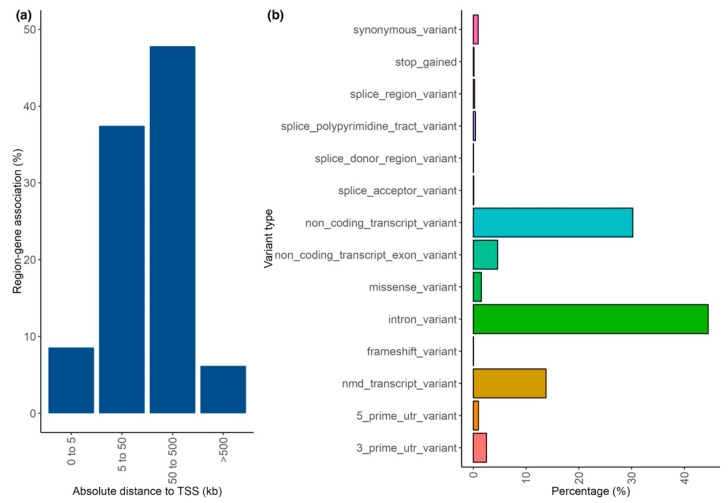
Annotation of genetic variants for asthma phenotypes. (**a**) Absolute distance (in kilobases) between genetic variants and the transcription start site (TSS) of the nearest gene; (**b**) variant effect type according to the Sequence Ontology term. Abbreviations: kb: kilobases; NMD: nonsense-mediated mRNA decay: TSS: transcription start site: UTR: untranslated region.

Most GWAS variants from asthma phenotype studies annotate to distal noncoding regions far from the transcription start site (TSS) of the closest gene (less than 500 kb). Functionally, most variants had consequences over introns, noncoding transcripts, or transcripts affected by nonsense-mediated mRNA decay (Figure 3a,b).

**Figure 4:**
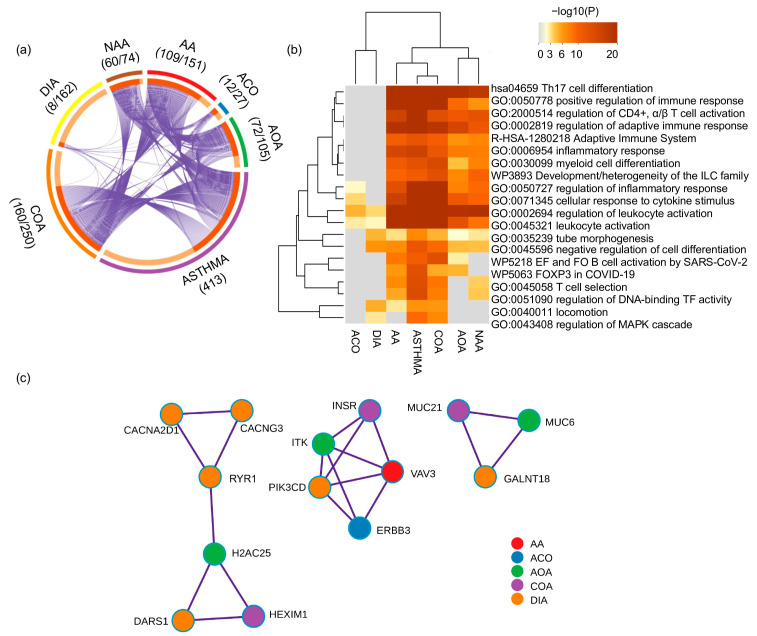
Genetic findings of asthma phenotypes. (**a**) Circos plot depicting the overlap of genes annotated based on the genetic variation associated across asthma phenotypes. The outer arc represents the identity of each gene list, whereas the inner arc represents a gene list, where each gene member of that list is assigned a spot on the arc. The dark orange color represents the genes shared by multiple gene lists, and light orange color represents genes specific to that gene list. The number of genes overlapping between each gene list and the 413 genes from the asthma gene list and the total number of genes within each gene list are shown along with each phenotype term. (**b**) Gene-set enrichment analysis of asthma and asthma phenotype-related genes. (**c**) Prioritized subnetworks for distinct genes for asthma phenotypes. Abbreviations: AA: allergic asthma; ACO: asthma–chronic obstructive pulmonary disease overlap syndrome; AOA: adult-onset asthma; COA: childhood-onset asthma; DIA: diisocyanate-induced asthma; EF: extrafollicular; FO: follicular; NAA: nonatopic asthma; TF: transcription factor.

COA and allergic asthma (AA) showed the highest gene overlap with asthma and cluster closely in terms of biological processes and pathways, while toluene diisocyanate-induced asthma (DIA) and ACO show the largest divergence (Figure 4a,b). The most shared terms in the enrichment analyses were related to inflammatory and adaptative immune responses (Figure 4b). A protein–protein interaction network of the most densely connected network components for asthma phenotypes prioritized three subnetworks (Figure 4c). The first subnetwork comprised four DIA, one COA, and one adult-onset asthma (AOA)-related genes implicated in calcium transport (*CACNG3*, *CACNA2D1*, and *RYR1*) or transcriptional/translational control (*DARS1*, *H2AC25,* and *HEXIM1*). The second subnetwork comprised five genes implicated in B-/T-cell receptor and PI3K/AKT signaling pathways (*ERBB3*, *ITK*, *INSR*, *PIK3CD*, and *VAV3*). The third subnetwork harbored three genes implicated in O-linked glycosylation of mucins (*GALNT18*, *MUC6*, and *MUC21*). None of these sub-networks contained NA-related genes.

### 3.2. Genomic Studies of Asthma-Related Traits

In recent years, great efforts have been made to characterize the genetic determinants of asthma treatment response and exacerbations, particularly in genetically admixed and historically minoritized populations disproportionately affected by asthma. Since genomic findings for asthma treatment response, asthma exacerbations, or gene–environment interactions have been recently reviewed elsewhere [85,86,87,88], this review will focus on age of asthma onset, moderate-to-severe asthma, asthma remission, T2-low asthma, as well as lung function, total IgE levels, and eosinophil-specific proteins in asthma.

Overall, 10 GWAS of asthma-related traits have been published (Table 2), primarily in European populations, except for GWAS of total serum and mite-specific IgE levels in East Asian individuals. Three GWAS [89,90,91] have investigated the association with age of asthma onset as a linear measurement. A GWAS meta-analysis of 5462 individuals with asthma and 8424 individuals without asthma [89] associated four stablished asthma loci with the age of asthma onset (2q12, 6p21, 9p24, and 17q12–q21) and found a novel association at locus 16q12. Moreover, in a GWAS including 37,846 patients with asthma [90], 19 loci were genome-wide associated with age of asthma onset, along with the genomic regions 2q12 and 9p24, previously detected [89]. Furthermore, a recent study [91] in 25,240 individuals with asthma uncovered novel significant genome-wide signals near genes implicated in the regulation of transcription (*TEF*), cell growth (*MUCL3*), and the prognosis of non-small-cell lung carcinoma (*SFTA2*) [92].

In terms of asthma severity, the largest genome-wide association study of moderate-to-severe asthma published to date identified three novel signals that regulate mucin production (rs10905284, rs11603634, and rs560026225) and validated 24 prior signals for mild asthma [93]. From the three novel signals, the SNP rs11603634 was specific to moderate-to-severe asthma. Moreover, a whole-genome sequencing association study of asthma severity in Europeans evidenced eight genome-wide significant loci previously reported as associated with asthma (*IL1RL2*, *TSLP*, *HLA-DQA1*, *BACH2*, *C11orf30*, *RAD51B*, and *GSDMB*) and lung function (*THSD4*) [94]. The inverse genetic correlation between moderate-to-severe asthma risk and lung function (r_g_ < −0.16) provides genetic support for a role of lung function in moderate-to-severe asthma risk. In this sense, only two GWAS of pulmonary function among individuals with asthma have been performed [95,96], focused mainly on Europeans. One study focused only on longitudinal FEV_1_ in children with asthma that received placebo and with data collected every 2–4 months in a 4-year period. Their work revealed seven suggestively associated SNPs that replicated in individuals that underwent nedocromil treatment (Table 1) [95]. Another study found replication for 7 out of 28 lung function loci (*HHIP*, *FAM13A*, *THSD4*, *GSTCD*, *NOTCH4-AGER*, *RARB*, and *ZNF323*) previously identified in the general population (*p*-value < 0.05) supporting a shared basis between phenotypes [96].

Although there are treatments to control asthma symptoms, as of today, there is no cure for asthma. Nonetheless, some individuals with asthma experience remission of their symptoms, which is more common in children than in adults [97]. The first and only GWAS of asthma remission to date [97] reported 25 SNPs suggestively associated in 790 Dutch adults (*p*-value < 2.5 × 10^−4^). Four of those associations were replicated in European adults (n = 1132). The top-hit, rs6581895, was found to be an expression quantitative trait locus (eQTL) of *FRS2* and *CCT2* in lung tissue. *FRS2* encodes a protein that belongs to the FRS2 family of adaptor/scaffold proteins and inhibits EGF signaling, which leads to an inhibition of EGF-induced cell proliferation and cell transformation. *CCT2* encodes a molecular chaperone which is part of the TCP1 ring complex (TRiC) and participates in the maintenance of cell proliferation. Genetic variants in these genes have been associated with albuminuria [98], which was recently associated with a greater decline in lung function [99]. Nevertheless, considering asthma as a risk factor for renal dysfunction remains controversial [100].

Nowadays, the inflammatory microenvironment in the lower airway remains unclear. Since the identification of biomarkers associated with T2 inflammation, one of the approaches used is to differentiate patients with T2-high from those who have T2-low asthma [20]. The only GWAS of T2-low adult asthma (n = 1350) revealed a genome-wide significant association (rs117639512, OR for A allele = 0.33, *p*-value = 2.75 × 10^−8^) in the intergenic region between kallikrein-related peptidase 4 (*KLK4*) and kallikrein-related peptidase 5 (*KLK5*) genes [101].

Although several GWAS have investigated genetic factors of IgE levels, only one GWAS of IgE levels in individuals with asthma has been conducted [102]. The analysis of 877 East Asians highlighted suggestive associations in *CRIM1*, *ZNF71*, *TLN1*, and *SYNPO2* that had not been previously associated with IgE in non-asthmatic individuals. However, these regions remain to be validated in independent studies to assess their potential interest as clinical markers. Notably, gene expression variation of *SYNPO2* has been previously associated with airway hyperresponsiveness in patients with asthma [103]. Similarly, although no GWAS of eosinophil counts has been conducted in asthma patients, two eosinophil-specific proteins released during allergic response have been studied in asthmatic families: ECP and eosinophil-derived neurotoxin (EDN) [104]. This study identified seven distinct signals located in five loci (1p31, 2p13, 7p21, 9q22, and 14q11) associated with ECP and EDN levels and/or the combination of both phenotypes in adults of asthma-ascertained families.

**Table 2:** Summary of independent genetic signals from GWAS of asthma-related traits.

Phenotypes	SNP	Chr. Region ^a^	Genomic Context	Effect Allele	Coefficient Type	Coefficient Value	*p*-Value	References
Asthma age onset	rs10208293	2q12	*IL1RL1*	G	HR	1.14	3.1 × 10^−8^	[89]
	rs9272346	6p21	*HLA-DQA1*	A	HR	1.13	1.6 × 10^−8^	
	rs928413	9p24	*IL33*	G	HR	1.19	6.5 × 10^−16^	
	rs1861760	16q12	*CYLD*	A	HR	1.28	4.2 × 10^−8^	
	rs9901146	17q12-q21	*ZPBP2/GSDMB*	G	HR	1.18	1.9 × 10^−16^	
	rs61816761	1q21.3	*FLG*	A	beta	−4.57	8.15 × 10^−27^	[90]
	rs7518129	1q25.1	*TNFSF4*	G	beta	−0.85	4.89 × 10^−9^	
	rs3771175	2q12.1	*IL1RL1*	T	beta	−1.73	7.66 × 10^−17^	
	rs10187276	2q36.3	*SNRPGP8/CCL20*	T	beta	−0.87	1.98 × 10^−8^	
	rs78147778	2q37.3	*D2HGDH*	T	beta	−0.91	1.64 × 10^−8^	
	rs2889896	3q28	*LPP*	C	beta	−0.98	8.07 × 10^−13^	
	rs5743618	4p14	*TLR1*	C	beta	−1.58	4.53 × 10^−22^	
	rs4705962	5q31.1	*KIF3A*	T	beta	−0.99	5.57 × 10^−10^	
	rs12207974	6p21.33	*HLA-E/RANP1*	C	beta	−1.07	8.86 × 10^−11^	
	rs1093	6p21.33	*HLA-B*	G	beta	−0.99	8.41 × 10^−10^	
	rs9274659	6p21.32	*HLA-DQB1/MTCO3P1*	A	beta	−1.25	6.71 × 10^−18^	
	rs7848215	9p24.1	*IL33*	T	beta	−1.03	7.51 × 10^−12^	
	rs117137535	9q34.3	*ARRDC1*	A	beta	−2.46	3.42 × 10^−8^	
	rs61894547	11q13.5	*EMSY*	T	beta	−2.23	4.42 × 10^−15^	
	rs12365699	11q23.3	*CXCR5/DDX6*	G	beta	−1.40	4.42 × 10^−14^	
	rs4795399	17q12	*GSDMB*	T	beta	−2.29	6.76 × 10^−65^	
	rs11658582	17q21.2	*CCR7/SMARCE1*	G	beta	−0.91	1.37 × 10^−10^	
	rs4574025	18q21.33	*TNFRSF11A*	T	beta	−0.87	1.61 × 10^−10^	
	rs12964116	18q21.33	*SERPINB7*	G	beta	−1.92	4.87 × 10^−8^	
	rs5758324	22q13.2	*TEF*	G	HR	1.06	2.39 × 10^−8^	[91]
	rs2844649	6p21.33	*SFTA2/MUCL3*	A	HR	1.08	4.45 × 10^−8^	
Asthma severity	rs560026225	4q27	*KIAA1109*	GATT	OR	1.12	3.06 × 10^−9^	[93]
	rs10905284	10p14	*GATA3*	A	OR	0.90	1.76 × 10^−10^	
	rs11603634	11p15.5	*MUC5AC*	G	OR	1.09	2.32 × 10^−8^	
	rs7523907	1q24.2	*CD247*	T	OR	1.10	4.82 × 10^−9^	
	rs12479210	2q12.1	*IL1RL1*	T	OR	1.19	1.57 × 10^−29^	
	rs34290285	2q37.3	*D2HGDH*	A	OR	0.84	2.24 × 10^−23^	
	rs1837253	5q22.1	*TSLP*	C	OR	1.19	1.95 × 10^−22^	
	rs1438673	5q22.1	*WDR36*	T	OR	0.89	3.29 × 10^−13^	
	rs3749833	5q31.1	*C5orf56*	C	OR	1.14	5.60 × 10^−14^	
	rs1986009	5q31.1	*RAD50*	A	OR	1.17	2.43 × 10^−15^	
	rs9273410	6p21.32	*HLA-DQB1*	A	OR	1.21	5.62 × 10^−32^	
	rs144829310	9p24.1	*IL33*	T	OR	1.21	2.29 × 10^−20^	
	rs7936312	11q13.5	*C11orf30*	T	OR	1.17	6.18 × 10^−24^	
	rs703816	12q13.3	*STAT6*	C	OR	1.12	3.69 × 10^−13^	
	rs10519068	15q22.2	*RORA*	A	OR	0.85	1.84 × 10^−12^	
	rs72743461	15q22.33	*SMAD3*	A	OR	1.14	4.52 × 10^−14^	
	rs7203459	16p13.13	*CLEC16A*	C	OR	0.86	4.37 × 10^−18^	
	rs2941522	17q12	*IKZF3*	T	OR	1.11	2.32 × 10^−12^	
	rs139210940	2q12.1	*IL1RL2*	AT	OR	1.34	8.08 × 10^−9^	[94]
	rs10455025	5q22.1	*TSLP*	C	OR	1.30	4.36 × 10^−13^	
	rs17205170	6p21.32	*HLA-DQA1*	G	OR	1.45	7.92 × 10^−16^	
	rs2875584	6q15	*BACH2*	C	OR	1.24	1.57 × 10^−8^	
	rs7130588	11q13.5	*C11orf30*	G	OR	1.24	2.46 × 10^−9^	
	rs2104047	14q24.1	*RAD51B*	T	OR	1.25	1.28 × 10^−8^	
	rs11631778	15q23	*THSD4*	G	OR	1.23	3.54 × 10^−8^	
	rs7216558	17q12	*GSDMB*	T	OR	1.26	1.91 × 10^−11^	
**Pulmonary function**								
FEV_1_	rs559389	11q13.4	*-*	C	beta	−0.03	5.28 × 10^−5^	[95]
	rs9366309	6p22.3	*-*	T	beta	−0.03	3.32 × 10^−5^	
	rs6763931	3q23	*ZBTB38*	A	beta	0.03	5.90 × 10^−5^	
	rs2304725	3p25.3	*SLC6A11*	C	beta	0.03	3.87 × 10^−5^	
	rs17161791	7p21.3	*-*	C	beta	0.002	3.01 × 10^−5^	
	rs10795348	10p13	*C10orf97*	T	beta	20.16	5.07 × 10^−5^	[96]
	rs10951730	7p13	*HECW1*	A	beta	20.24	9.58 × 10^−5^	
	rs1291183	18p11.32	*YES1*	T	beta	20.18	3.54 × 10^−5^	
	rs1321267	6q23.2	*MOXD1*	A	beta	20.14	5.46 × 10^−5^	
	rs1843593	4q13.2	*GNRHR*	T	beta	20.20	5.30 × 10^−5^	
	rs2040403	22q12.3	*SYN3*	A	beta	0.29	1.96 × 10^−5^	
	rs2063485	3q13.11	*ZPLD1*	T	beta	0.23	7.18 × 10^−5^	
	rs285461	1q23.3	*LRRC52*	T	beta	0.22	9.56 × 10^−5^	
	rs3010301	6q24.1	*CITED2*	T	beta	0.17	2.65 × 10^−5^	
	rs3756089	4q35.1	*IRF2*	T	beta	0.26	8.79 × 10^−5^	
	rs3805383	4q12	*NMU*	A	beta	20.17	2.35 × 10^−5^	
	rs388159	19p13.11	*IL12RB1*	T	beta	0.19	3.47 × 10^−5^	
	rs4234121	2q37.3	*KIF1A*	A	beta	20.15	3.25 × 10^−5^	
	rs4651208	1q25.3	*C1orf21*	T	beta	20.15	6.34 × 10^−5^	
	rs4735916	8p23.3	*ERICH1*	A	beta	20.24	7.02 × 10^−5^	
	rs5755023	22q12.3	*LARGE*	A	beta	20.21	1.75 × 10^−5^	
	rs58667	22q13.31	*UPK3A*	A	beta	0.18	3.95 × 10^−7^	
	rs6788848	3q13.11	*ZPLD1*	T	beta	20.18	4.99 × 10^−5^	
	rs7434819	4q32.1	*C4orf18*	A	beta	20.14	7.34 × 10^−5^	
	rs7670758	4q31.21	*HHIP*	A	beta	20.14	9.50 × 10^−5^	
	rs7836170	8q13.2	*SULF1*	T	beta	20.16	2.15 × 10^−5^	
	rs925847	2q32.2	*STAT4*	T	beta	0.16	8.17 × 10^−5^	
	rs9364299	6q27	*SMOC2*	A	beta	0.14	9.96 × 10^−5^	
	rs9903394	17q24.3	*SOX9*	A	beta	0.16	8.47 × 10^−5^	
FVC	rs6482071	10p12.31	*BCL11A*	T	beta	20.16	6.15 × 10^−5^	
	rs2497714	10q25.3	*ALS2CR4*	T	beta	20.17	8.49 × 10^−5^	
	rs2181563	10q25.3	*TNIK*	A	beta	0.20	3.50 × 10^−5^	
	rs10466868	12q24.33	*YTHDC1*	T	beta	0.35	1.39 × 10^−5^	
	rs6500728	16p13.3	*KIAA0922*	T	beta	20.17	2.02 × 10^−5^	
	rs169660	16p12.2	*SULF1*	T	beta	20.17	9.77 × 10^−5^	
	rs1291183	18p11.32	*ABLIM1*	A	beta	0.33	4.66 × 10^−5^	
	rs11085898	19p13.12	*PLXDC2*	T	beta	0.31	6.50 × 10^−5^	
	rs2110565	2p16.1	*KIAA1600*	A	beta	20.17	6.72 × 10^−5^	
	rs1208082	2q33.1	*LOC338797*	T	beta	20.25	6.39 × 10^−5^	
	rs221013	20p12.2	*HS3ST2*	T	beta	20.17	4.20 × 10^−5^	
	rs8115491	20q12	*A2BP1*	T	beta	20.17	7.15 × 10^−5^	
	rs6096573	20q13.2	*YES1*	T	beta	20.18	5.47 × 10^−5^	
	rs9974012	20q13.31	*NDUFB7*	A	beta	0.16	4.97 × 10^−5^	
	rs7281703	21q21.1	*PAK7*	T	beta	0.17	3.09 × 10^−5^	
	rs8140240	22q12.3	*ATP9A*	T	beta	0.16	5.92 × 10^−5^	
	rs4133045	3q26.2	*PTPRT*	T	beta	0.20	7.02 × 10^−5^	
	rs17592868	4q13.2	*BMP7*	T	beta	0.16	8.72 × 10^−5^	
	rs13119846	4q31.3	*C21orf37*	T	beta	0.33	6.98 × 10^−5^	
	rs7836170	8q13.2	*EIF3S7*	A	beta	20.31	9.95 × 10^−5^	
FEV_1_/FVC	rs11032873	11p13	*APIP*	T	beta	0.18	3.08 × 10^−5^	
	rs11675728	2q36.3	*DNER*	T	beta	20.15	7.22 × 10^−5^	
	rs12659620	5p15.31	*ADCY2*	T	beta	0.15	5.56 × 10^−5^	
	rs1406593	7p15.2	*SNX10*	T	beta	20.15	7.64 × 10^−5^	
	rs1416920	6p22.1	*ZNF323*	T	beta	0.16	9.46 × 10^−5^	
	rs17450685	10q22.3	*C10orf11*	T	beta	0.15	8.73 × 10^−5^	
	rs17554448	2q31.1	*ZNF650*	A	beta	0.19	8.56 × 10^−5^	
	rs17646998	8q13.2	*SULF1*	T	beta	20.15	3.61 × 10^−5^	
	rs2063485	3q13.11	*ZPLD1*	T	beta	0.25	1.34 × 10^−5^	
	rs2230739	16p13.3	*ADCY9*	A	beta	0.16	6.25 × 10^−5^	
	rs2705044	8p22	*MTMR7*	A	beta	0.21	7.46 × 10^−5^	
	rs3130696	6p21.33	*HLA-C*	A	beta	20.17	7.34 × 10^−5^	
	rs3748540	1q43	*GREM2*	A	beta	0.15	7.88 × 10^−5^	
	rs3809335	13q12.13	*MTMR6*	T	beta	0.28	5.40 × 10^−5^	
	rs4234121	2q37.3	*KIF1A*	A	beta	20.15	5.32 × 10^−5^	
	rs5767064	22q13.32	*LOC388915*	A	beta	0.16	2.79 × 10^−5^	
	rs7663065	4p15.1	*FLJ45721*	A	beta	20.15	5.43 × 10^−5^	
	rs8030494	15q24.1	*TBC1D21*	A	beta	0.15	7.83 × 10^−5^	
	rs823673	1p34.2	*NFYC*	A	beta	0.19	3.94 × 10^−5^	
	rs9287995	2q31.1	*HNRPA3*	T	beta	0.15	6.02 × 10^−5^	
	rs9362054	6q14.3	*C6orf84*	T	beta	0.14	8.68 × 10^−5^	
	rs9574386	13q31.1	*C13orf10*	A	beta	20.27	7.72 × 10^−5^	
**Asthma remission**								[97]
Clinical remission	rs7240102	18q21.2	*LOC100130003/C18orf26*	G	OR	1.99	7.9 × 10^−5^	
Complete remission	rs6581895	12q15	*YEATS4/FRS2*	G	OR	3.83	1.3 × 10^−5^	
	rs12405429	1q42.2	*FAM89A/TRIM67*	G	OR	3.10	1.0 × 10^−4^	
	rs1420101	2q12.1	*IL18R1/IL1RL1*	A	OR	0.44	3.4 × 10^−4^	
T2-low asthma	rs117639512	19q13.41	*KLK5*	A	OR	0.33	2.75 × 10^−8^	[101]
Total IgE	rs10404342	19q13.43	*ZNF71*	C	NA	NA	7.60 × 10^−6^	[102]
	rs4879926	9p13.3	*TLN1*	C	NA	NA	7.74 × 10^−60^	
**Eosinophil-specific proteins**								[104]
EDN	rs72677651	1p31.3	*JAK1/AK4*	T	beta	0.54	2.0 × 10^−8^	
	rs76335186	7p21.3	*NDUFA4*	G	beta	−0.55	4.9 × 10^−8^	
	rs67049014	14q11.2	*RNASE2/METTL17*	A	beta	−0.32	3 × 10^−12^	
ECP	rs56675562	9q22.1	*CDK20/SPATA31C2*	G	beta	−0.56	5.1 × 10^−9^	
ECP-EDN	rs116571378	2p13.3	*ARHGAP25*	T	beta	NA	4.2 × 10^−10^	
	rs67049014	14q11.2	*RNASE2/METTL17*	A	beta	NA	1 × 10^−13^	

^a^ Positions based on GRCh37/hg19 build. Abbreviations: FEV1: forced expiratory volume in 1 second; FVC: forced vital capacity; HR: hazard ratio; IgE: immunoglobulin E; NA: not available; OR: odds ratio.

## 4. Discussion

Our analysis of the NHGRI-EBI GWAS Catalog highlighted a Eurocentric bias in studies of asthma phenotypes and related traits, as similarly observed for GWAS across human diseases/traits [105] and polygenic scores [106]. Further efforts should be made in ethnically diverse populations, particularly in historically minoritized populations disproportionately affected by asthma susceptibility, mortality, and comorbidities, such as African Americans and Hispanics/Latinos in the United States of America. In this regard, recent studies have characterized the genetic variation implicated in asthma exacerbations and treatment response in admixed populations either by GWAS [85,86], multi-omic approaches [107], or leveraging local ancestry via admixture mapping [108,109,110]. However, functional studies are needed to confirm these findings and prioritize genetic markers for assessment of their predictive capability to guide treatment response or prognosis.

Some of the genes revealed in GWAS of asthma phenotypes and related traits have been also uncovered in non-stratified GWAS of asthma. Therefore, it is likely that many of the previously reported asthma signals actually reflect specific asthma phenotypes present in a large proportion of GWAS participants. Although several studies have focused on asthma phenotypes, there is a limited number of studies considering only asthma patients, especially for clinically relevant phenotypes such as lung function or IgE levels, which could also include healthy individuals. For instance, novel IgE-related genes (*CRIM1*, *ZNF71*, *TLN1*, and *SYNPO2*) were uncovered by the only GWAS of IgE in asthma patients. Among these, *SYNPO2* gene overexpression was previously associated with reduced airway hyperresponsiveness in individuals with asthma after oral corticoid therapy [103]. Another study focused on eosinophil-specific proteins released during allergic response [104] revealed genetic signals in genes involved in pathophysiologic mechanisms common between eosinophil activity and asthma, such as inflammation, oxidative stress, and extracellular matrix remodeling. The research on asthma remission merits special attention, despite the fact that just one study has been conducted so far [97]. In their work, the A-allele of rs1420101 located in *IL1RL1* was associated with a lower probability of complete asthma remission in adults. This finding is consistent with a previous study that reported the association of this SNP with higher eosinophil levels in childhood asthma and with a higher risk for asthma [111]. All these together support the necessity of considering asthma-related phenotypes as a strategy to potentiate novel loci discovery and possible new therapeutical targets for precision medicine. Although the characterization of genetic influences on asthma phenotypes still lags behind compared to other omic layers (e.g., proteomics [112], transcriptomics [93,113,114], or epigenetics [115,116]), three studies have discerned asthma-related polygenic phenotypes [117,118,119]. The analysis of comorbidity data from electronic health records as a surrogate of unknown gene–environment contexts distinguished 22 asthma subgroups with distinct comorbidity patterns using approximately six million residents of the United States of America, from which 11 subgroups were validated in the UKB [117]. The GWAS of asthma across the validated subgroups and the whole dataset revealed 14 shared and 6 distinct associations, of which loci for the musculoskeletal and gastrointestinal asthma subgroups remained significant after stringent correction for multiple testing [117]. An analysis of longitudinal data from the UKB revealed multiple age-dependent comorbidity subgroups across complex diseases. In particular, the asthma subgroup characterized by dermatological comorbidities exhibited significant heterogeneity in polygenic risk scores compared to the other asthma subgroups [118]. Another study found suggestive genetic associations for asthma-related phenotypes determined by latent class analysis of clinical and demographic data from 3001 European adults [119].

Other approaches for identification of polygenic subtypes for human complex diseases and traits [120] could be implemented in asthma. Although the effect size gradient is likely to be small for most asthma-related loci [117], clustering of variant effect sizes supported by functional annotation or pleiotropy may also reveal additional insights into the genetic basis of respiratory diseases. Multi-trait genetic analyses of autoimmune and/or allergic diseases have uncovered pleotropic variants in European [121,122] and Japanese individuals [123]. Conversely, the modest differences in minor allele frequencies of most common variants across subgroups may hinder genomic-driven subtype identification in complex diseases [120]. A recent analysis in the UKB overcame this limitation by investigating previous respiratory-health-related loci and incorporating multi-trait data into genetic effect clustering by considering airway diseases, lung function, and other clinical and demographic traits [124]. The evident Eurocentric bias in genomic research of respiratory diseases is likely to lessen with the development of large-scale initiatives integrating both genomic, environmental, and respiratory health data from ethnically diverse populations, such as the Environmental influences on Child Health Outcomes (ECHO) study [125] and the All of Us Research Program [126]. Moore et al. [16] used a clustering approach in the SARP cohort to classify severe asthma cases attending to clinical and demographic data and were able to discriminate several sub-phenotypes within this cohort of patients.

Multi-omics have also been applied to define asthma phenotypes and gain a better comprehension of the disease. For instance, Forno et al. prioritized *IL5RA* as a candidate gene associated with asthma using vertical integration of several analytical layers [127]. Another potential strategy to integrate several omic layers could be the use of colocalization [128]. In this case, each layer is analyzed separately and then overlapped to evaluate if there are specific genomic regions that associate with a trait of interest through more than one omic layer, implying and reinforcing the association of that region with the phenotype studied. In that regard, it is important that the tissues to be explored are relevant for the trait under study since the transcriptomic and epigenetic profiles may differ between tissues. Nonetheless, these strategies have been scarcely applied to define asthma subgroups or to assess any asthma phenotype individually.

Importantly, one omic layer rarely included in asthma multi-omic analyses despite the amount of linking evidence with asthma development, progression, and asthma exacerbations is the microbiome, defined as the set of microorganisms of a specific niche, which gathers both the human microbiome and the environmental microbiome. In fact, several studies have assessed the implication of the bacterial and fungal communities of the airways and related tissues as the saliva or the oral cavity, highlighting how dysbiosis in those environments associates with risk or protection of developing asthma or asthma exacerbations [129].

An alternative to these integrative methods could be the exploration of the role that genetic variants highlighted in omic studies as associated with a specific trait have in the context of transcriptomic and epigenetic regulation or other contexts such as the RNA maturation or the tridimensional organization of the genetic material in the nucleus (histone modification and DNA packing). These methods have been collectively called quantitative trait locus (QTL) analyses and have been recently incorporated in omic studies to allocate those associations in the cellular context and gain a better understanding with regard to the biological meaning of those associations [130]. An important note for future studies concerns the annotation methodology used to assign genes to genetic variants in order to understand omic results from a functional perspective and as part of a bigger picture. Since this annotation method assigns genes to genetic variants attending to proximity to the TSS, a plausible alternative for future studies could be using annotation methods that rely on functional information instead of only relying on physical distance.

## Figures and Tables

**Table 1:** Overview of asthma endotypes and phenotypes.

Endotype	Phenotype	Clinical Features	Molecular Mechanism	References
T2-high asthma	Early-onset atopic asthma	Trigger-induced phenotypes. Steroid-sensitive. Preserved lung function	Allergy to aeroallergens	[8,13,14,19,25,26,27,28,29]
	Late-onset eosinophilic asthma	CRSwNP frequently associated. Steroid-refractory	*Staphilococcus aureus* enterotoxin	[11,12,30,31,32]
	NERD	Samter´s Syndrome. Adult onset. Trigger-induced phenotypes	Arachidonic acid dysregulation	[33,34,35,36,37,38,39,40]
T2-low asthma	Non-atopic asthma	Neutrophilic or paucigranulocytic.	Th1/Th17 inflammation	[22,41,42,43,44,45,46,47,48,49,50,51,52,53,54]
		Adult onset		
	Smoking-associated asthma	Adult onset. Lower lung function	Oxidative stress	[62,63,64,65,66,67]
	Obesity-associated asthma	Metabolic syndrome. Females. Preserved lung function	Th1/Th17 inflammation. Oxidative stress. IL-6	[55,56,57,58,59,60,61]
	Elderly-related asthma	Very late onset. Declined lung function	Th1/Th17 inflammation	[70,71,72,73,74]

## Data Availability

All data necessary to evaluate the conclusions of this manuscript are available in the NHGRI-EBI GWAS Catalog and reported in the main text and/or Appendix A.

## Appendix A

### Appendix A.1. Literature and Database Mining

The NHGRI-EBI GWAS Catalog (version 2023-04-07) was downloaded from the GWAS catalog webpage and processed in R 4.2.3. The GWAS Catalog literature mining, inclusion criteria, and curation process is described elsewhere [82,131]. The methodology for categorization of individuals into ancestry groups is detailed elsewhere [132]. Ancestry groups were categorized into seven groups: “African” (AFR), “African American or Afro-Caribbean” (AA/AC), “Asian” (ASN), “European” (EUR), “Hispanic/Latin American” (LAT), “Other/Mixed” (O/M), “Not Reported (NR)”. Outcomes containing the words “asthma” or “Asthma” in the disease/category term were categorized into six groups according to the type of assessed genetic effect: (a) main effects for asthma, (b) main effects for asthma and other diseases/traits (pleiotropy), (c) effects of gene–environment or gene–gene interactions, (d) main effects for asthma subtypes, or (e) main effects for asthma-related phenotypes. Diseases/traits related to asthma were grouped according to the following seven categories: asthma, asthma–chronic obstructive pulmonary disease overlap syndrome (ACO), nonatopic asthma (NAA), allergic asthma (AA), adult-onset asthma (AOA), childhood-onset asthma (COA), and toluene diisocyanate-induced asthma (DIA). Asthma-related diseases/traits were grouped according to the following six categories: asthma remission, control, and severity, age of asthma onset, immunoglobulin E levels, eosinophil-specific protein levels, bronchial hyperresponsiveness, and lung function. Asthma exacerbations, treatment response, and gene–environment interactions on asthma were considered for scientometric analysis but were not reviewed as they have been recently described in depth [85,86,87]. The full list of phenotypes considered for scientometric purposes is detailed in Table A1.

**Table A1:** Categorization of diseases/traits for asthma phenotypes and related traits available in the NHGRI-EBI GWAS Catalog (version 2023-04-07).

Asthma-Related Traits	Asthma Phenotype
**AES**	**AA**
Asthma exacerbations	Atopic asthma
Asthma with exacerbation (PheCode 495.2)	Asthma and hay fever
Childhood asthma exacerbations in long-acting beta2-agonist treatment	Asthma and eczema
Childhood asthma with severe exacerbations	ICD10 J45.0: Predominantly allergic asthma
Severe exacerbations in childhood asthma	ICD10 J45.0: Predominantly allergic asthma (Gene-based burden)
Exacerbations requiring hospitalization in asthma	**ACO**
Asthma exacerbations in inhaled corticosteroid treatment	Asthma–COPD overlap syndrome
Asthma with severe exacerbations	Asthma–chronic obstructive pulmonary disease overlap syndrome in asthma
**Age of onset**	Asthma–COPD overlap syndrome (Gene-based burden)
Asthma (time to childhood onset) in early life tobacco smoke exposure)	Asthma–chronic obstructive pulmonary disease overlap syndrome
Asthma (time to onset)	**AOA**
Asthma (age of onset)	Adult-onset asthma in non-smokers
Age of onset of childhood-onset asthma	Adult-onset asthma in ever-smokers
Age of onset of adult-onset asthma	Adult asthma (Gene-based burden)
Asthma (time to event)	Asthma (adult onset)
**CSR**	Adult asthma
Asthma (moderate or severe)	**COA**
Clinical remission in asthma	Pediatric asthma
Complete remission in asthma	Self-reported childhood asthma in adult smokers
Asthma control	Asthma (childhood onset)
**GxE**	Childhood asthma
Asthma (sex interaction)	Asthma onset (childhood vs. adult)
Adult onset asthma (smoking interaction)	**DIA**
Childhood asthma x sex interaction	Asthma (toluene diisocyanate-induced)
Asthma (SNP x SNP interaction)	Diisocyanate-induced asthma
Asthma control x inhaled corticosteroid treatment interaction (1df)	**NAA**
Asthma control x inhaled corticosteroid treatment interaction (2df)	Nonatopic asthma
Asthma x Hispanic interaction (2df)	
Bronchodilator response x age interaction in asthma	
Bone mineral accretion in asthma (oral corticosteroid dose interaction)	
Response to zileuton treatment in asthma (FEV1 change interaction)	
Asthma (time to childhood onset) x early life tobacco smoke interaction	
Adult onset asthma (smoking interaction)	
Asthma (sex interaction)	
Asthma (SNP x SNP interaction)	
Asthma or atopy (farm exposure interaction)	
Asthma x air pollution interaction (2df)	
Asthma x Hispanic interaction (2df)	
Childhood asthma x sex interaction	
Childhood onset asthma (traffic air pollution exposure interaction)	
Lung function (FEV1) in asthma (dust mite allergen exposure interaction)	
Lung function (FEV1/FVC) in asthma (dust mite allergen exposure interaction)	
Adverse response to inhaled corticosteroid treatment x age interaction in asthma	
Bronchodilator response in asthma (inhaled corticosteroid treatment interaction)	
Post-bronchodilator FEV1 x air pollution (CO) interaction in childhood asthma)	
Asthma x air pollution interaction (2df)	
Post-bronchodilator FEV1 x air pollution (NO2) interaction in childhood asthma,	
Childhood onset asthma (traffic air pollution exposure interaction)	
**IgE-related**	
IgE levels in asthmatics	
IgE levels in asthmatics (D.f. specific)	
IgE levels in asthmatics (D.p. specific)	
**Lung function**	
Pulmonary function in asthmatics,	
Lung function (FEV1) in asthma	
Lung function (FVC) in asthma	
Lung function (FEV1/FVC) in asthma	
**Treatment response**	
Post-bronchodilator lung function in asthma (FEV1)	
Post-bronchodilator lung function in asthma (FVC)	
Post-bronchodilator lung function in asthma (FEV1/FVC)	
Asthma treatment response	
Asthma (bronchodilator response)	
Asthma (corticosteroid response)	
Response to inhaled corticosteroid treatment in asthma (change in FEV1)	
Response to inhaled glucocorticoid treatment in asthma (change in FEV1)	
Bronchodilator response in asthma	
Response to montelukast in asthma (change in FEV1)	
Response to mepolizumab in severe asthma	
Response to placebo treatment in childhood asthma (FVC change)	
Oral corticosteroid burst in asthma	
Recent medication for asthma (UKB data field 22167)	
Recent medication for asthma (UKB data field 22167) (Gene-based burden)	
Subjective response to placebo treatment in childhood asthma (change in cough/wheeze)	

Abbreviations: 2df: 2 degree of freedom test; AA: allergic asthma; ACO: asthma–chronic obstructive pulmonary disease overlap syndrome; AEs: asthma exacerbations; AOA: adult-onset asthma; CO: carbon monoxide; COA: childhood-onset asthma; COPD: chronic obstructive pulmonary disease; CSR: asthma control, severity, or remission; Dp: *Dermatophagoides pteronyssinus;* Df: *Dermatophagoides farinae*; DIA: diisocyanate-induced asthma; FEV1: forced expiratory volume in 1 s; FVC: forced vital capacity; GxE: gene–environment interaction; IgE: immunoglobulin E; NAA: nonatopic asthma; NO2: nitrogen dioxide; SNP: single-nucleotide polymorphism; UKB: United Kingdom Biobank.

To identify GWAS studies that have yet to be included in the NHGRI-EBI GWAS Catalog, Pubmed was queried using the following command via PubmedR R package [133] in order to retrieve studies from inception up to 19 April 2023: “((((((Genome-Wide Association Study[MeSH Terms]) AND (asthma[MeSH Terms])) AND (asthma[Title])) NOT (exacerbations[Title])) NOT (editorial[Publication Type])) NOT (Review[Publication Type])) NOT (Systematic Review[Publication Type])”. Screening of articles was conducted independently by two reviewers (A.E.-O. and E.H.-L.). Any disputes in data were resolved in a joint meeting between the two reviewers (A.E.-O. and E.H.-L.). Conference or poster abstracts, literature reviews, editorials, or opinion articles, studies not conducted on humans, studies not in English, and studies not designed as genome-wide association were excluded. One article with genome-wide significant signals for COA was identified and incorporated into the database. In addition, we further conducted a systematic search using Pubmed to identify genomic studies for asthma remission, asthma control, asthma severity, age of asthma onset, immunoglobulin E levels, eosinophils count, eosinophil-specific protein levels, bronchial hyperresponsiveness, and lung function. No genomic studies of eosinophils count in asthma have been conducted.

### Appendix A.2. Variant Annotation

Genetic variants were annotated to genes via GREAT [134] using the “basal plus extension” procedure, which considers proximal genes as those with a transcription start site (TSS) within 5.0 and 1.0 kilobases (kb) upstream and downstream of the genomic location, respectively, and distal genes as those with a TSS located up to 1000 kb from the genomic location. Variant effect type according to the Sequence Ontology term was investigated using g:SNPsense within the g:Profiler framework [135]. Multi-effect variants were considered in the analysis.

### Appendix A.3. Enrichment and Protein–Protein Interaction Network Analyses

Gene-set enrichment analyses were conducted for genes detected only by the specific parent categories or shared across categories using default parameters at Metascape (v3.5.2023-05-01) [136]. The following terms were evaluated using gene-set enrichment analysis on Gene ontology terms (GO) for Cellular Components, Molecular Functions, and Biological Processes, as well as additional pathway data, including the Kyoto Encyclopedia of Genes and Genomes (KEGG) Pathway, Reactome Gene Sets, Canonical Pathways, and WikiPathways datasets. Within the Metascape framework, a protein–protein interaction network was built considering distinct gene sets for asthma phenotypes and physical interactions in STRING [137] and BioGrid [138]. The Molecular Complex Detection (MCODE) algorithm [139] was used to identify densely connected network components.
